# Activation of the Alternate Renin-Angiotensin System Correlates with the Clinical Status in Human Cirrhosis and Corrects Post Liver Transplantation

**DOI:** 10.3390/jcm8040419

**Published:** 2019-03-27

**Authors:** Stephen Casey, Robert Schierwagen, Kai Yan Mak, Sabine Klein, Frank Uschner, Christian Jansen, Michael Praktiknjo, Carsten Meyer, Daniel Thomas, Chandana Herath, Robert Jones, Jonel Trebicka, Peter Angus

**Affiliations:** 1 Liver Unit, Austin Health, Melbourne 3084, Australia; robert.jones@austin.org.au; 2 Department of Medicine, Austin Health, University of Melbourne, Melbourne 3084, Australia; Kai.Mak@mh.org.au (K.Y.M.); cherath@unimelb.edu.au (C.H.); 3 Department of Internal Medicine I, University of Bonn, 53113 Bonn, Germany; robert.schierwagen@kgu.de (R.S.); Sabine.klein@kgu.de (S.K.); frank.uschner@kgu.de (F.U.); Christian.jansen@ukbonn.de (C.J.); Michael.praktiknjo@ukbonn.de (M.P.); 4 Department of Internal Medicine I, Goethe University Clinic Frankfurt, 60323 Frankfurt, Germany; 5 Victorian Infectious Disease Reference Laboratory, the Peter Doherty Institute for Infection and Immunity, Melbourne Health, Melbourne 3000, Australia; 6 Institute of Cellular Medicine, Fibrosis Research Group, Newcastle upon Tyne NE2 4HH, UK; 7 Department of Radiology, University of Bonn, 53113 Bonn, Germany; Carsten.meyer@ukbonn.de (C.M.); Daniel.thomas@ukbonn.de (D.T.); 8 Institute for Bioengineering of Catalonia, 08028 Barcelona, Spain; 9 European Foundation for the Study of Chronic Liver Failure, 08021 Barcelona, Spain; 10 Department of Gastroenterology and Hepatology, Odense University Hospital, 5000 Odense, Denmark

**Keywords:** cirrhosis, portal hypertension, renin-angiotensin system

## Abstract

Introduction: Recent animal studies have shown that the alternate renin-angiotensin system (RAS) consisting of angiotensin-converting enzyme 2 (ACE2), angiotensin-(1–7) (Ang-(1–7)) and the Mas receptor is upregulated in cirrhosis and contributes to splanchnic vasodilatation and portal hypertension. To determine the potential relevance of these findings to human liver disease, we evaluated its expression and relationship to the patients’ clinical status in subjects with cirrhosis. Methods: Blood sampling from peripheral and central vascular beds was performed intra-operatively for cirrhotic patients at the time of liver transplantation (LT) or trans-jugular intra-hepatic portosystemic shunt (TIPS) procedures to measure angiotensin II (Ang II) and Ang-(1–7) peptide levels and ACE and ACE2 enzyme activity. Relevant clinical and hemodynamic data were recorded pre-operatively for all subjects and peripheral blood sampling was repeated 3 months or later post-operatively. Results: Ang-(1–-7) and ACE2 activity were up-regulated more than twofold in cirrhotic subjects both at the time of LT and TIPS and levels returned to comparable levels as control subjects post-transplantation. Ang-(1–7) levels correlated positively with the degree of liver disease severity, as measured by the model for an end-stage liver disease (MELD) and also with clinical parameters of pathological vasodilatation including cardiac output (CO). There were strong correlations found between the ACE2:ACE and the Ang-(1–7):Ang II ratio highlighting the inter-dependence of the alternate and classical arms of the RAS and thus their potential impact on vascular tone. Conclusions: In human cirrhosis, the alternate RAS is markedly upregulated and the activation of this system is associated strongly with features of the hyperdynamic circulation in advanced human cirrhosis.

## 1. Introduction

Vasodilatation of the splanchnic circulation plays a pivotal role in the development of portal hypertension and the hyperdynamic circulatory state in human cirrhosis [1]. The mechanisms responsible for pathological vasodilatation in advanced cirrhosis are not fully understood. It is clear that there is an increase in the production of the endothelial-derived circulatory vasodilator, nitric oxide (NO), in the splanchnic vascular bed [2,3,4]. However, there is evidence that a number of other mechanisms are also involved including the increased production of other endogenous vasodilators and impairments of compensatory vasoconstriction responses [5,6,7].

In cirrhosis, there is known to be upregulation of the ‘classical’ renin-angiotensin system (RAS) and increased production of its key effector peptide, Ang II. This occurs locally within the liver, where it contributes to liver fibrosis [8] and also systemically where activation occurs in an attempt to maintain blood pressure and the central filling in response to vasodilatation and a reduced effective arterial blood volume [9,10,11]. Our understanding of the RAS has evolved in recent times following the discovery of an ‘alternate’ counter-regulatory arm of the RAS. Within this alternate arm, the angiotensin-converting enzyme 2 (ACE2), a homolog of ACE expressed by the vascular endothelium and liver parenchymal cells, [12,13,14] degrades Ang II generating the Ang-(1–7) peptide [13,14,15]. This peptide exerts vasodilatory and anti-fibrotic effects [11,15] via the Mas receptor, a G protein-coupled receptor [16]. Ang-(1–7), in turn, can then be degraded by the ACE enzyme to the biologically inactive peptide Ang-(1–5) [17]. This ‘alternate axis’ is thought to act primarily as a counter-regulatory arm protecting tissues from potentially harmful effects of classical RAS activation [18,19,20].

Recent studies from our laboratory have shown that the alternate RAS is upregulated in chronic liver disease with the increased expression of ACE2 resulting in increased production of Ang-(1–7) in both the liver and circulation being observed in animal models of cirrhosis [11,13,21]. Furthermore, in a group of cirrhotic patients at liver transplantation, the portal Ang-(1–7):Ang II ratio was found to correlate negatively with systemic vascular resistance [22] leading to the hypothesis that activation of this alternate vasodilatory arm of the RAS may contribute to the circulatory changes known to occur in human cirrhosis. Moreover, our group has most recently shown that in experimental cirrhosis and portal hypertension upregulated ACE2 in the cirrhotic mesenteric vascular bed increased the production of the vasodilator Ang-(1–7) from Ang II breakdown and Ang-(1–7), in turn, markedly reduced vascular tone in the mesenteric bed of cirrhotic animals [15]. These findings suggest that activation of the alternate arm of the RAS may play an important role in mediating vasodilatation in cirrhosis.

In order to further explore the concept of alternate RAS activation and its potential impact upon vascular pathophysiology in human cirrhosis, we measured the circulatory components of the contemporary RAS and important clinical parameters in a large cohort of cirrhotic patients who were undergoing either liver transplantation or a trans-jugular intra-hepatic portosystemic shunt (TIPs) procedure.

## 2. Experimental Section

### 2.1. Study Subjects

Patients known to have cirrhosis who were listed for liver transplantation (LT group, *n* = 25) or due to undergo a TIPS procedure were recruited for the study. Patients who underwent TIPS were sub-grouped into those who took non-selective beta-blockers (NSBB TIPS group, *n* = 54) as secondary prevention for variceal bleeding and those that did not (TIPS group, *n* = 35). This distinction between groups was made due to the fact that NSBB therapy has been shown to attenuate RAS activation [23]. A healthy cohort (*n* = 15) of subjects undergoing gastrointestinal endoscopy procedures without known liver disease or portal hypertension were also recruited. Significant liver disease was excluded in the controls subjects on the basis of history and clinical examination. Participants were excluded from the study if they had co-morbidities (i.e., cardiovascular disease, pulmonary or renal disease) or took medications other than NSBBs known to affect the RAS. Patients with cirrhosis were taking a range of medication including spironolactone, furosemide and antibiotics for SBP prophylaxis whilst control subjects were on no medications. Informed written consent was obtained from all subjects prior to study recruitment. The study adhered to the principles of the Declaration of Helsinki and was approved by the local human research ethics committees.

### 2.2. Blood Sampling

Blood sampling at liver transplantation was performed initially from the portal vein (10 mL) by the surgical team after the exposure of the vessel and prior to vessel clamping. Immediately afterward, sampling was performed from a peripheral artery (10 mL) and the right atrium (10 mL) via catheters inserted for routine hemodynamic monitoring during the procedure. During the TIPS procedures, blood sampling (10 mL per region) was performed from a peripheral vein and the portal vein prior to the creation of the portosystemic shunt. In the control subjects, blood sampling (10 mL) was performed from a peripheral vein only. A number of patients in the LT (*n* = 15) and TIPS (*n* = 6) groups had a follow up peripheral venous sample (10 mL) taken at 3 months or more after their surgery.

Plasma obtained for ACE and ACE2 activity analysis was collected in ice-cold lithium heparin tubes whilst plasma for the measurement of angiotensin peptides was collected in ice-cold tubes containing an inhibitor mix (N-ethylmalaimide 0.2 M, Na_2_EDTA 50 μM, aprotonin 21,000 U/mL, leupeptin 10.5 μM and pepstatin-A 1.5 μM) to prevent breakdown by circulating peptidases. Samples were centrifuged (2250 g force for 15 min at 4 °C) and the plasma was separated and stored at −20 °C for later analysis.

### 2.3. Measurement of ACE and ACE2 Activity and Angiotensin Peptide Levels

Plasma ACE activity was measured by spectrophotometric assessment of ACE mediated substrate cleavage as previously described [11]. A quenched fluorescent substrate (QFS), which comprised a fluorophore, 7-methoxy- coumarin-4-acetyl (MCA) and a quencher, N-2,4-dinitrophenyl separated by a short peptide chain (alanine, proline and lysine), was used to assess ACE2 activity. The measurement was performed by incubating subject samples with the QFS substrate and recording fluorescence generated by the cleavage reaction at excitation and emission wavelengths of 320 nm and 405 nm, respectively. [14] A fluorescence plate reader (FLUOstar Optima, BMG LABTECH, Germany) was used for this purpose. The rate of substrate cleavage was then determined by comparison to a standard curve of the free fluorophore, MCA (Sigma Aldrich, Australia). Plasma Ang-(1–7) and Ang II levels were quantified using direct radioimmunoassay as previously described by our group (Prosearch International, Australia) [13].

### 2.4. Hemodynamic Parameters

The mean arterial pressure (MAP) and heart rate (HR) were recorded for all study participants pre-operatively. Cirrhotic subjects in the study also had a non-invasive assessment of CO determined by the left ventricular outflow tract doppler trans-thoracic echocardiography method shown to demonstrate acceptable agreement with the gold standard measurement of CO by thermodilution method [24,25].

### 2.5. Data and Statistical Analysis

Results are expressed as mean ± standard error of the mean (SEM). Data were analysed using Student’s t-test and Spearman’s correlation test and repeated measures ANOVA (where appropriate) followed by Tukey’s multiple comparison test. *p* < 0.05 was considered statistically significant.

## 3. Results

### 3.1. Patient Characteristics

The demographics of the study participants are displayed in Table 1. The aetiologies of liver disease in the LT group included hepatitis C virus (HCV, *n* = 9), primary sclerosing cholangitis (PSC, *n* = 5), non-alcoholic fatty liver disease (NAFLD, *n* = 4), hepatitis B virus (HBV, *n* = 2), alpha 1 anti-trypsin deficiency (A1A-T, *n* = 2), primary biliary cirrhosis (PBC, *n* = 2) and alcohol (*n* = 1). Patients in the TIPS group included alcohol (*n* = 19), HCV (*n* = 8), A1A-T (*n* = 1), Budd-Chiari syndrome (*n* = 1) and cryptogenic liver disease (*n* = 6). The NSBB TIPS group comprised of patients affected by the following aetiology of liver disease: alcohol (*n* = 31), HCV (*n* = 8), NAFLD (*n* = 2), Budd-Chiari syndrome (*n* = 2) A1A-T (*n* = 1), auto-immune hepatitis (*n* = 1) and cryptogenic liver disease (*n* = 9).

The LT group had the more advanced liver disease than both TIPS groups as evidenced by their higher model for end-stage liver disease (MELD) and Child-Pugh scores (Table 1). The LT group also had clinical features of a more vasodilated circulation with a higher CO than other cirrhosis groups. Both CO and HR were lower in the NSBB TIPS group compared to other cirrhosis groups. 

### 3.2. Peripheral Ang-(1–7), Ang II and ACE2 Levels are Elevated in Human Cirrhosis

**Figure 1:**
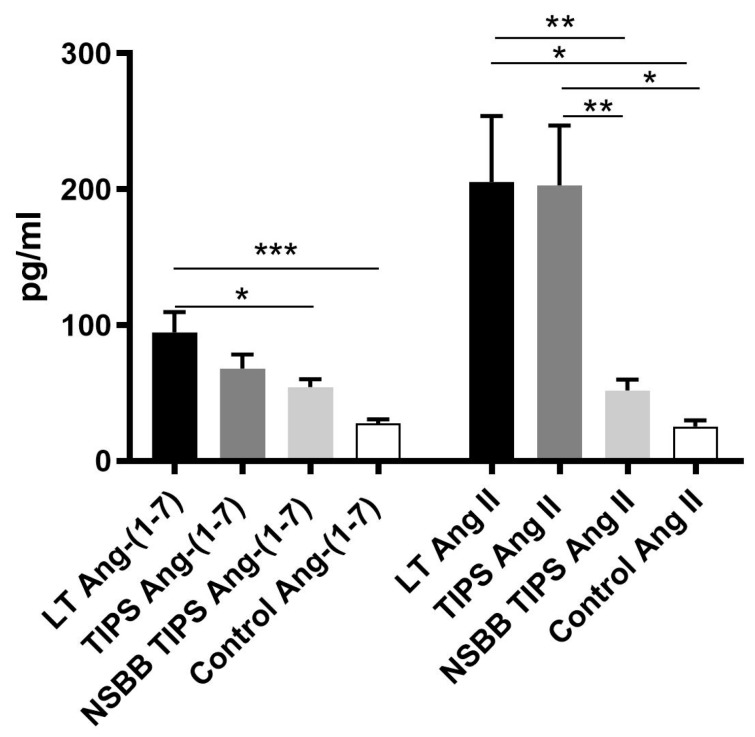
The peripheral measurement of circulating angiotensin-(1–7) and angiotensin II. Angiotensin-1–7 (Ang-(1–7)) was significantly elevated in the liver transplantation (LT) group compared to the control group and among cirrhotic subjects, the Ang-(1–7) levels were higher in the LT group compared to the non-selective beta-blocker (NSBB) trans-jugular intra-hepatic portosystemic shunt (TIPS) group. Angiotensin II (Ang II) was significantly elevated in the LT and TIPS groups compared to both the NSBB TIPS and control groups. * *p* < 0.05, ** *p* < 0.01, *** *p* < 0.001.

Ang-(1–-7) levels measured in the peripheral circulation were significantly elevated in the LT group compared to the control subjects (Figure 1), and in the TIPS group, there was a trend toward a significant elevation of Ang-(1–7) when compared to controls (*p* = 0.0679). Among cirrhotic subjects, Ang-(1–7) levels were higher in the LT group compared to the NSBB TIPS group (Figure 1). As expected, in cirrhotic groups (LT and TIPS) there was evidence of the counter-regulatory activation of vasoconstrictor responses with marked elevation (up to tenfold) of peripheral levels of the vasoconstrictor Ang II (Figure 1) [7,11], however, the degree of Ang II upregulation was significantly attenuated in the NSBB TIPS group, as has previously been shown [23], to the extent that Ang II levels in this group were not statistically different to the control group. This finding thus suggests that NSBB therapy may play an inhibitory role in the RAS activation in human cirrhosis. 

**Figure 2:**
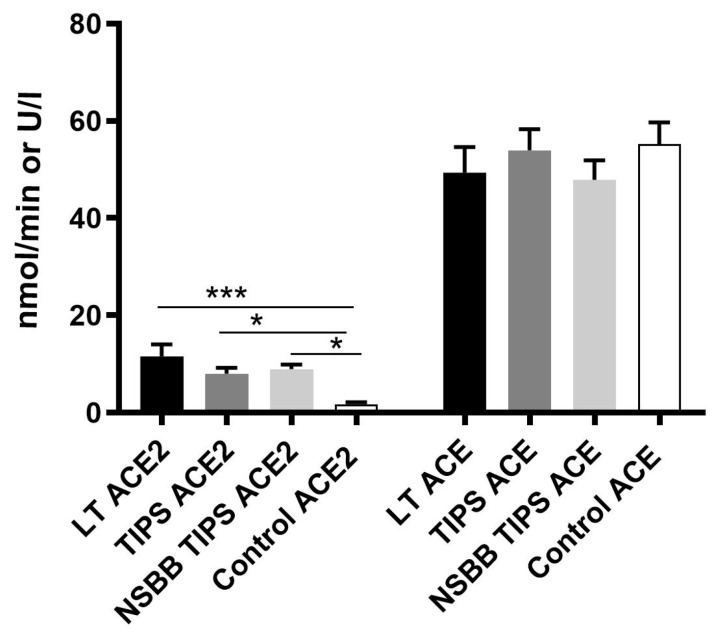
The peripheral measurement of circulating angiotensin-converting enzyme 2 (ACE2) and ACE enzyme activity. ACE2 enzyme activity was significantly elevated in the LT, TIPS and NSBB TIPS groups compared to the control group. ACE enzyme activity was comparable between all groups. * *p* < 0.05, *** *p* < 0.001.

Peripheral ACE2 enzyme activity was also up-regulated at least four-fold in the LT and both TIPS groups compared to controls (Figure 2). However, the ACE enzyme activity did not differ between cirrhotic groups and control subjects (Figure 2). 

### 3.3. Ang-(1–7), Ang II and ACE2 Levels Return to Normal Post Liver Transplantation

**Figure 3:**
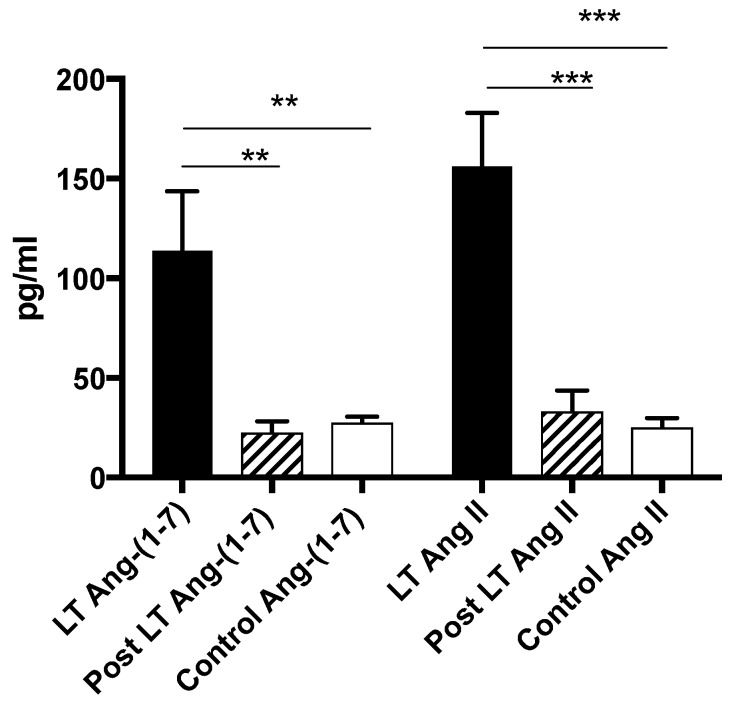
The peripheral measurement of circulating angiotensin-(1–7) and angiotensin II levels in LT subjects. Ang-(1–7) and Ang II levels in the peripheral circulation were elevated in LT subjects but returned to comparable levels as controls post liver transplantation. ** *p* < 0.01, *** *p* < 0.001.

**Figure 4:**
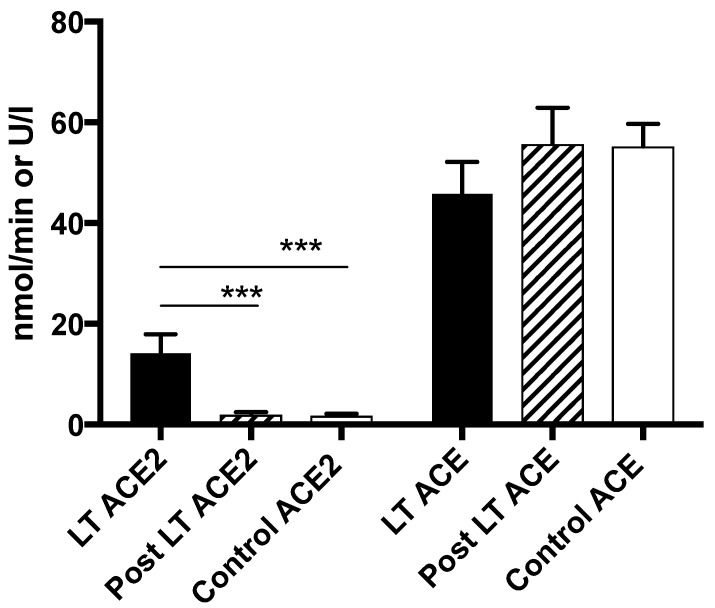
The peripheral measurement of circulating ACE2 and ACE enzyme activity in LT subjects. ACE2 enzyme activity measured in the peripheral circulation was elevated in LT subjects but returned to comparable levels as controls post liver transplantation. ACE enzyme activity was comparable across all groups. *** *p* < 0.001.

Post liver transplant, with presumed normalization of hemodynamics [26,27], peripheral levels of Ang-(1–7), Ang II and ACE2 activity all returned to similar levels as controls in the LT group (Figure 3 and Figure 4) suggesting the deactivation of both arms of the RAS. In contrast to the LT group, however, there were no significant changes in any measured RAS parameter in the small no of patients from the TIPS group (6/35) who had post TIPS blood sampling.

### 3.4. Comparison of Peptide and ACE2 Enzyme Activity Levels in Different Vascular Beds

In the LT group, the Ang II level was lowest at the portal vein and highest at the radial artery whilst Ang-(1–7) levels were similar throughout regions (Table 2). A likely explanation for the elevated levels of Ang II found at the radial arterial bed is that the peripheral arterial circulation is directly downstream from the ACE rich pulmonary circulation [28]. In keeping with this finding, the ratio of Ang-(1–7):Ang II was higher in the portal circulation than the peripheral arterial circulation, as has been previously found in human subjects at the time of liver transplantation (Table 2) [22]. ACE2 activity was similar in all regions. 

In the TIPS group (not on NSBB therapy) Ang II was also lower in the portal compared to the peripheral circulation, however, this did not reach statistical significance. All other measured RAS parameters were similar between regions in this group (Table 3).

Ang-(1–7) was elevated in the portal circulation in comparison to the peripheral circulation of NSBB TIPS subjects whilst ACE2 activity and Ang II were comparable between measured regions (Table 4). As previously described (Figure 1), the NSBB TIPS group had a significantly lower level of Ang II in the peripheral circulation and this was also the case for the portal circulation when compared to the TIPS group (Table 3 and Table 4) despite the fact that these groups had similarly advanced liver disease with comparable ACE activity. This finding may be explained by the inhibitory effect of beta-blocker therapy on renin secretion [29]. An alternative or contributory explanation to consider, however, may be an increased rate of Ang II peptide degradation via the ACE2 enzyme which was found to be elevated in the portal circulation of the NSBB TIPS group compared to the TIPS group (*p* = 0.0018) (Table 3 and Table 4.) 

### 3.5. The Relative RAS Enzyme Activity (ACE2:ACE) is Highly Predictive of Local Peptide Production

In the LT group, a positive relationship was found between Ang-(1–7) and ACE2 enzyme activity in every vascular bed sampled (Table 5). Furthermore, there were strong positive correlations between ACE2:ACE and the level of Ang-(1–7) whilst even stronger correlations were seen between ACE2:ACE and the Ang-(1–7):Ang II ratio (Table 5). It is also noteworthy that in the peripheral vascular bed of LT subjects (radial artery) where Ang II levels were highest, an inverse correlation was found between ACE2:ACE and Ang II. Similarly, a positive correlation between ACE2:ACE and the Ang-(1–7):Ang II ratio was observed in the TIPS (peripheral bed *p* = 0.002, *r* = 0.595) and NSBB TIPS (portal vein *p* = 0.003, *r* = 0.505) groups. No such significant correlations were found for the control subjects.

### 3.6. Ang-(1–7) is Increased in Cirrhotic Patients with Advanced Liver Disease and Clinical Features of a Hyperdynamic Circulation

There was a significant relationship between liver disease severity (as assessed by MELD) and Ang-(1–7) in the LT group in bloods taken from the portal vein (Table 6). In all vascular beds of the LT group, there were positive correlations between Ang-(1–7) and CO, suggesting greater alternate RAS up-regulation in cirrhotics with a more vasodilated circulation (Table 6). Similarly, in the TIPS group, positive correlations were found between Ang-(1–7) levels and MELD score, HR and CO (Table 7). 

### 3.7. Alternate RAS Upregulation is Greatest in Patients with Ascites

Abdominal ascites is a clinical hallmark of a more advanced stage of portal hypertension and its development is associated with marked cardiovascular, renal and neuro-humoral changes [30,31]. There were 16 patients in the LT group who required treatment for ascites prior to transplant and 9 patients without clinical ascites (Table 8). As expected, the ascitic sub-group had a more advanced form of the liver disease as determined by their higher MELD and Child-Pugh scores. The ascitics also had stronger features of a hyperdynamic circulation with a strong trend towards higher CO in keeping with the greater vasodilatation in this sub-group (Table 8).

**Figure 5:**
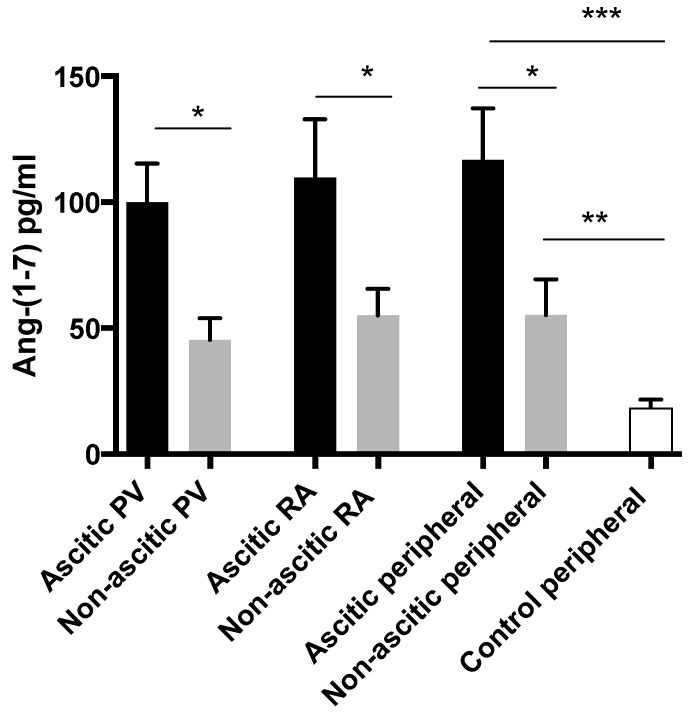
The regional measurements of circulating angiotensin-(1–7) in ascitic and non-ascitic LT subjects. Ang-(1–7) was elevated in ascitic patients compared to non-ascitics in all regions sampled, * *p* < 0.05, ** *p* < 0.01, *** *p* < 0.001. PV = portal vein, RA = right atrium.

In patients undergoing liver transplantation, circulating levels of Ang-(1–7) were higher in ascitic cirrhotics compared to non-ascitic cirrhotics in all three regions (portal vein, right atrium and peripheral) sampled (Figure 5). Although Ang-(1–7) levels were lower in non-ascitic cirrhotics, they were still significantly higher than in control subjects when peripheral levels were compared. Angiotensin II levels did not differ between ascitic and non-ascitic subjects in any sampled region but, as expected, both cirrhotic sub-groups had higher levels than controls.

**Figure 6:**
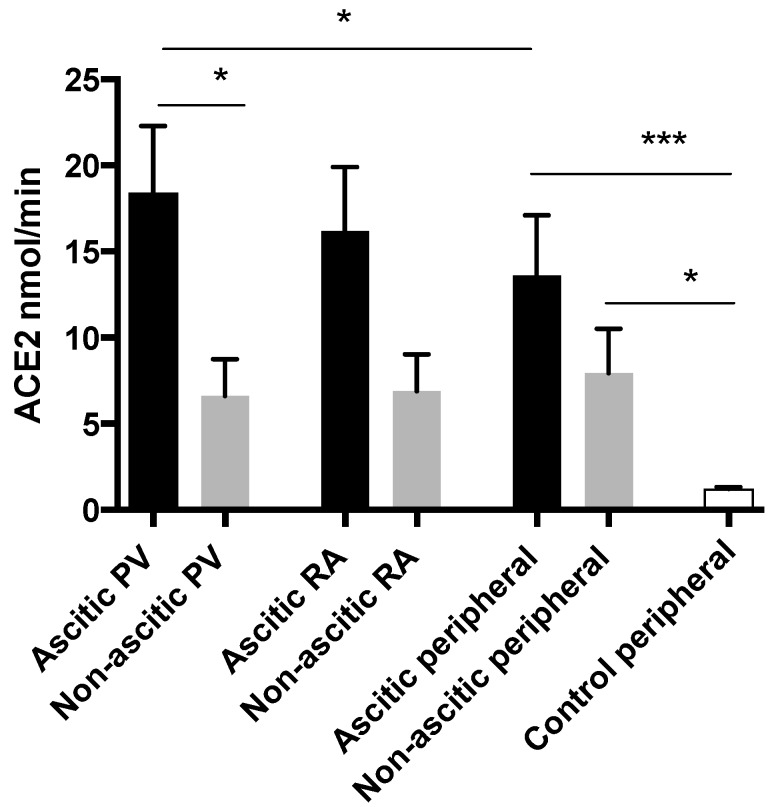
The regional measurements of ACE2 activity in ascitic and non-ascitic LT subjects. ACE2 enzyme activity was elevated in ascitic patients compared to non-ascitics when measured in the portal circulation. Among ascitic cirrhotics in the LT group, ACE2 was highest in the portal circulation, * *p* < 0.05, *** *p* < 0.001. PV = portal vein, RA = right atrium.

In keeping with the Ang-(1–7) levels, portal venous ACE2 enzyme activity was also greater in the ascitic sub-group than those without ascites (Figure 6). In the peripheral circulation, ACE2 levels were higher in both cirrhotic sub-groups compared to controls. Among the ascitic sub-group, ACE2 activity in the portal circulation was significantly higher than when measured peripherally.

## 4. Discussion

This study reveals for the first time that both ACE2 and Ang-(1–7) of the alternate RAS are markedly up-regulated in human cirrhosis but that the activation of the alternate renin-angiotensin system returns to normal post liver transplantation when the circulatory abnormality associated with advanced cirrhosis has corrected [27,32]. Importantly, there were strong positive correlations between Ang-(1–7) levels and CO as measured by echocardiography suggesting that the levels are greatest in subjects with a more vasodilated circulation, thus supporting the hypothesis that activation of the alternate RAS contributes to vasodilation in cirrhosis.

Activation of the alternate RAS was also associated with a more advanced form of the liver disease as determined by MELD score. The ascitic sub-group of liver transplantation patients had the most advanced liver disease and vasodilated circulations of all study subjects and, among these patients, ACE2 activity was highest in the splanchnic circulation from where pathological vasodilation in cirrhosis originates [33,34]. These findings are thus consistent with animal studies where the upregulation of the mesenteric alternate RAS was shown to contribute to the reduced mesenteric vascular tone in experimental cirrhosis [15].

Similarly to previous studies, Ang II levels were increased in both cirrhotic patients at liver transplantation surgery and in patients undergoing TIPS [7,11]. In comparison, cirrhotic subjects taking NSBB therapy, however, had marked downregulation of Ang II levels suggesting that NSBBs may have a role to play in RAS deactivation through an as-yet-unknown mechanism. Ang II levels returned to normal post liver transplantation with the restoration of normal circulatory function. In contrast, in the TIPS group, where only portal blood flow had been altered, high levels of Ang II persisted post procedure, indicating that there was ongoing RAS activation in these cirrhotic patients in an attempt to maintain blood pressure and renal perfusion [9,34]. As has been previously documented in human cirrhosis, Ang II levels in the portal circulation were lower in both the LT and TIPS groups compared to levels measured peripherally [22]. One possible explanation for this finding may be a shift in the splanchnic vascular bed RAS expression and activity towards the alternate arm, resulting in the increased Ang II peptide degradation by upregulated ACE2 enzyme activity. However, the portal ACE2 activity was comparable to that measured in the peripheral circulation and the Ang-(1–7) levels did not vary between regions in the LT group. An alternative explanation for the Ang II imbalance between splanchnic and peripheral circulations is the closer proximity of the peripheral circulation to the ACE rich pulmonary circulation which is the primary site of Ang II synthesis [28,35]. Thus, areas of the circulation directly downstream of the lungs would be expected to have the highest Ang II levels.

Both the ACE2 and ACE enzymes are a product of the vascular endothelium [10,35] and are critical to the generation of Ang-(1–7) and Ang II peptides where ACE2 breaks down Ang II to produce Ang-(1–7) whilst ACE generates Ang II from Ang I and degrades Ang-(1–7) [13,17]. The finding in this study of a strongly positive correlation between ACE2:ACE and Ang-(1–7):Ang II ratios in both the liver transplant and TIPS cirrhotic groups is consistent with the concept that the relative levels of Ang-(1–7) and Ang II are dictated by the relative activities of these two enzymes. Importantly, the ratio of Ang-(1–7): Ang II could, in turn, have an important influence on local vascular tone [22].

It is important to note that post-transplant samples were obtained from a peripheral vein whilst peri-transplant peripheral samples were taken from the radial artery. However, when samples from the peripheral arterial and venous circulations (taken simultaneously) of cirrhotic TIPS subjects were compared, no significant differences in respective peptide and enzyme values were found between these 2 vascular beds. In addition, in the healthy controls, the peptide and enzyme values taken from either a peripheral artery or vein were also comparable. Thus it is unlikely that the marked fall in Ang-(1–7) and ACE2 levels post-transplant was due to a change in the peripheral site from which the bloods were taken.

In conclusion, this study supports the previous findings in animal work from our laboratory and others, suggesting that the alternate RAS is activated in cirrhosis leading to increased circulating levels of the vasodilator peptide, Ang-(1–7) [15]. The degree of upregulation of the alternate RAS appears to be greatest in the portal circulation and in patients with clinical features of a vasodilated circulation. Deactivation of the alternate RAS occurs post liver transplantation with the restoration of normal circulatory function. Further studies should now be undertaken to determine the relevance of these findings to human liver disease by examining vascular responses of humans to Ang-(1–7) in vivo.

## Figures and Tables

**Table 1:** The subject demographics and clinical data.

	LT (*n* = 25)	TIPS (*n* = 35)	NSBB TIPS (*n* = 54)	Control (*n* = 15)
**Age**	53 (2)	53 (2)	61 (2) *	50 (4)
**Sex (M/F)**	20/5	26/9	31/23	7/8
**Child Pugh score**	10 (0.5) *	7 (0.3)	7 (0.2)	N/A
**MELD**	21 (2) *	12 (1)	12 (1)	N/A
**MAP**	84 (2)	80 (2)	80 (2)	78 (2)
**HR**	83 (3)	83 (3)	73 (2) #	64 (2) !
**CO**	6.9 (0.4) *	5.4 (0.4) ^	3.4 (0.3)	N/A

* *p* < 0.05 vs all other groups; # *p* < 0.05 vs other cirrhosis groups; ! *p* < 0.05 vs all cirrhosis groups; ^ *p* < 0.05 vs NSBB TIPS group. LT, liver transplantation; TIPS, trans-jugular intra-hepatic portosystemic shunt; MELD, model for an end-stage liver disease; MAP, mean arterial pressure; HR, heart rate; CO, cardiac output.

**Table 2:** The regional levels of RAS components in liver transplant (LT) subjects (* *p* < 0.05, ** *p* < 0.01 vs. radial artery).

	Portal Vein	Right Atrium	Radial Artery
**Ang-(1–7) pg/mL**	81(12)	90 (17)	95 (16)
**Ang II pg/mL**	138 (24) **	163 (30)	206 (52)
**Ang-(1–7):Ang II**	1.022 (0.18) *	1.194 (0.34)	0.828 (0.18)
**ACE2 nmol/L**	14.1 (2.8)	13.6 (2.9)	12.1 (2.7)

**Table 3:** The regional levels of RAS components in TIPS subjects.

TIPS	Portal Vein	Peripheral Vein	*p*-Value
**Ang-(1–7) pg/mL**	64 (11)	68 (10)	NS
**Ang II pg/mL**	119 (27)	175 (46)	NS
**Ang-(1–7):Ang II**	0.65 (0.1)	0.74 (0.1)	NS
**ACE2 nmol/L**	6.5 (1)	8 (1)	NS

**Table 4:** The regional levels of RAS components in NSBB TIPS subjects.

NSBB TIPS	Portal Vein	Peripheral Vein	*p*-Value
**Ang-(1–7) pg/mL**	65 (7)	55 (6)	0.0148
**Ang II pg/mL**	53 (9)	50 (8)	NS
**Ang-(1–7):Ang II**	1.59 (0.2)	1.32 (0.2)	0.068
**ACE2 nmol/L**	10 (1)	9 (1)	NS

**Table 5:** The regional correlation analysis in LT subjects (RAS parameters). PV = portal vein, RA = right atrium, Rad art = radial artery. Numerical values in black and red font denote the *p*-value and Spearman correlation coefficient (*r*) respectively. Brackets indicate an inverse correlation.

	Local Ang-(1–7)	Local Ang II	Local 1–7:II
**PV ACE2**	0.004 0.590	0.433	0.003 0.611
**PV ACE**	0.41	0.64	0.345
**PV ACE2:ACE**	0.007 0.56	0.195	<0.0001 0.7
**RA ACE2**	0.004 0.557	0.869	0.008 0.515
**RA ACE**	0.657	0.17	0.471
**RA ACE2:ACE**	0.006 0.536	0.27	0.00024 0.671
**Rad art ACE2**	0.044 0.406	0.092 (0.344)	<0.0001 0.711
**Rad art ACE**	0.759	0.183	0.341
**Rad art ACE2:ACE**	0.031 0.432	0.035 (0.423)	<0.0001 0.8

**Table 6:** The regional correlation analysis in LT subjects (clinical parameters). PV = portal vein, RA = right atrium, Rad art = radial artery. Numerical values in black and red font denote the *p*-value and Spearman correlation coefficient (*r*), respectively.

	MELD	Child-Pugh	CO	MAP	HR
**PV** **Ang-(1–7)**	0.042 0.428	0.057 0.403	0.004 0.648	NS	NS
**RA** **Ang-(1–7)**	0.108	NS	0.003 0.635	NS	NS
**Rad art** **Ang-(1–7)**	NS	NS	0.015 0.537	NS	NS

**Table 7:** The regional correlation analysis in the TIPS group (clinical parameters). PV = portal vein, Periph = peripheral vein. Numerical values in black and red font denote the *p*-value and Spearman correlation coefficient (*r*), respectively.

	MELD	Child-Pugh	CO	MAP	HR
**PV** **Ang-(1–7)**	0.059 0.338	NS	0.002 0.632	NS	0.032 0.379
**Periph** **Ang-(1–7)**	0.032 0.399	NS	0.07 0.232	NS	0.075 0.336

**Table 8:** The demographics of ascitic and non-ascitic LT sub-groups.

	Ascitic (*n* = 16)	Non-Ascitic (*n* = 9)	*p*-Value
**Age**	52 (2)	55 (3)	NS
**Sex (M/F)**	13/3	7/2	NS
**MELD**	24 (2)	16 (3)	<0.05
**Child Pugh score**	11 (0.5)	8 (1)	<0.05
**MAP**	84 (3)	84 (4)	NS
**HR**	85 (3)	81 (5)	NS
**CO**	7.4 (0.5)	5.9 (0.4)	0.0612
